# Norepinephrine Versus Dopamine as Initial Vasoactive Therapy in Neonatal Septic Shock: A Randomized Controlled Trial in a Tertiary Care Neonatal Intensive Care Unit in Eastern India

**DOI:** 10.7759/cureus.114127

**Published:** 2026-08-07

**Authors:** Moparthi Puramjai, Deepti D Pradhan, Santosh K Panda, Soumini Rath, Nirmal K Mohakud, Subhra Snigdha Panda, Manas K Nayak

**Affiliations:** 1 Pediatrics, Kalinga Institute of Medical Sciences, Bhubaneswar, IND; 2 Pediatrics, Akkineni Hospitals, Vijayawada, IND; 3 Pediatric Medicine, Kalinga Institute of Medical Sciences, Bhubaneswar, IND; 4 Medical Microbiology, Kalinga Institute of Medical Sciences, Bhubaneswar, IND

**Keywords:** dopamine, neonates, noradrenaline, sepsis, shock

## Abstract

Background

Septic shock remains a major cause of neonatal morbidity and mortality, particularly in preterm and low birth weight neonates. Although dopamine was traditionally used as the first-line vasoactive agent in neonatal shock, norepinephrine has emerged as a potential alternative agent of late because of its potent vasoconstrictive effects and favourable hemodynamic profile. However, evidence comparing the efficacy of these agents in septic shock in neonates remains limited.

Objective

To compare the efficacy of norepinephrine and dopamine as the initial vasopressor in neonatal septic shock.

Methods

This study was a non-blinded parallel randomized controlled trial conducted between October 2020 and October 2022 in the neonatal intensive care unit of a tertiary care center in Odisha, India. Ninety neonates with septic shock were randomized into two groups: norepinephrine group (n=45) and dopamine group (n=45). Norepinephrine was initiated at 0.2 mcg/kg/min, while dopamine was started at 10 mcg/kg/min with escalation according to clinical response. The primary outcome was reversal of shock within 45 minutes of initiation of vasoactive therapy. Secondary outcomes included requirement of additional vasoactive drugs, need for mechanical ventilation, all-cause mortality by 28 days, and incidence of complications including intraventricular hemorrhage (IVH), bronchopulmonary dysplasia (BPD), necrotizing enterocolitis (NEC), and retinopathy of prematurity (ROP).

Results

Baseline demographic and clinical characteristics of both groups were comparable. Reversal of shock at 45 minutes was achieved in 14 neonates (31.1%) in the norepinephrine group and 16 neonates (35.5%) in the dopamine group (p=0.65). Hemodynamic parameters including systolic blood pressure, diastolic blood pressure, and capillary refill time improved progressively in both groups without statistically significant intergroup differences. Requirement of additional vasoactive support was observed in 11 neonates (24.4%) in the norepinephrine group and 10 neonates (22.2%) in the dopamine group (p=0.80). Mechanical ventilation was required in 16 neonates (35.5%) in the norepinephrine group and 14 neonates (31.1%) in the dopamine group (p=0.65). All-cause mortality was comparable between the groups (15.5% vs. 13.3%; p=0.76). The incidence of short-term complications including IVH, NEC, BPD, and ROP was also similar between groups.

Conclusion

Norepinephrine and dopamine demonstrated comparable efficacy in achieving early reversal of neonatal septic shock, with no significant differences in mortality or short-term complications. Both agents may be considered suitable initial vasoactive therapies in neonatal septic shock. Larger multicentric studies are required to establish definitive evidence-based recommendations for vasoactive management in this population.

## Introduction

In spite of recent advances in neonatal intensive care and antimicrobial therapy, neonatal septic shock is still a major cause of morbidity and mortality worldwide, particularly in resource-limited settings [1,2]. Neonatal sepsis contributes substantially to neonatal deaths, especially among preterm and very low birth weight infants [3]. The pathophysiology of septic shock in neonates is complex and multifactorial, involving vasoregulatory dysfunction, myocardial impairment, altered microcirculation, and inadequate tissue perfusion leading to multiorgan dysfunction [4-6]. Early recognition and prompt hemodynamic stabilization are crucial for improving neonatal outcomes [5,6].

Initial management of neonatal septic shock includes fluid resuscitation; however, many neonates subsequently develop fluid-refractory shock requiring vasoactive support [7,8]. Dopamine has traditionally been used as the first-line vasoactive agent because of its combined inotropic and vasoconstrictive properties [8]. However, dopamine use has been associated with inconsistent hemodynamic responses and adverse effects such as tachyarrhythmias and endocrine dysfunction [9,10].

Norepinephrine has recently emerged as a potential alternative vasopressor in neonatal septic shock due to its potent α-adrenergic vasoconstrictive effect, resulting in improved systemic vascular resistance and stabilization of blood pressure [11,12]. Previous neonatal studies have demonstrated favourable hemodynamic effects with norepinephrine use in distributive shock states [11-13]. Nevertheless, evidence comparing dopamine and norepinephrine in neonatal septic shock remains limited and inconsistent [14,15].

Current neonatal septic shock guidelines do not clearly recommend a single superior first-line vasoactive agent because of limited high-quality neonatal evidence [5-7]. Furthermore, most of the previous studies done on initial vasopressor supports in neonates are limited by small sample sizes, heterogeneous study populations, and lack of standardized hemodynamic monitoring [14,16].

Therefore, this randomized controlled trial was done to compare the efficacy of norepinephrine and dopamine as the initial vasopressor in neonatal septic shock. The primary objective was to assess reversal of shock within the first 45 minutes after initiation of therapy. Our study also aimed to evaluate the baseline acid-base status and metabolic characteristics of neonates presenting with septic shock upon admission. Secondary objectives included need of additional vasoactive drugs and mechanical ventilation, all-cause mortality by 28 days of life and occurrence of complications such as intraventricular hemorrhage (IVH), necrotizing enterocolitis (NEC), bronchopulmonary dysplasia (BPD) and retinopathy of prematurity (ROP) until discharge.

## Materials and methods

This non-blinded parallel randomized controlled trial was conducted over a period of two years from October 2020 to October 2022 in the neonatal intensive care unit (NICU) of a tertiary care center in Odisha, India, after Institutional Ethics Committee approval and registration with the Clinical Trials Registry of India (CTRI/2021/01/030785). Written informed parental consent was obtained prior to enrollment of all participants.

All admitted neonates in the NICU with clinical features suggestive of sepsis and shock were eligible for inclusion. Neonates with congenital heart disease, multiple congenital malformations, hypoxic ischemic encephalopathy, or those requiring surgical intervention were excluded from the study.

Shock was defined by the presence of either hypotension, i.e., systolic blood pressure or diastolic blood pressure below the 5th percentile for gestational age or the presence of at least two or more of the following clinical signs - tachycardia (>160/minute), capillary refill time (CRT) over three seconds, and core-to-peripheral temperature difference >3°C [5,6]

Sepsis evaluation included complete blood count (CBC), C-reactive protein (CRP), blood culture, urine culture, and cerebrospinal fluid (CSF) analysis. CSF findings suggestive of meningitis included CSF cell count >30 cells/mm³, protein >150 mg/dL, and/or glucose <40 mg/dL [17].

Gestational age was determined using maternal last menstrual period, first-trimester ultrasonography, or modified New Ballard scoring [18]. Baseline neonatal characteristics of birth weight, gestational age, mode of delivery, Apgar score at five minutes, signs and symptoms of sepsis, and sepsis screening parameters were recorded. Maternal characteristics including antenatal steroid administration, premature rupture of membranes (PROM), and maternal hypothyroidism were also documented [19].

Baseline hemodynamic assessment included temperature, heart rate, respiratory rate, blood pressure, CRT and arterial blood gas analysis. Blood pressure was monitored noninvasively using a multiparameter monitor (Philips Medical Systems, Inc., Andover, MA, USA). Peripheral temperature was measured using a Hicks digital thermometer (Hicks Thermometers India Ltd., Aligarh, India), while core temperature was assessed rectally by a Philips monitor. Additionally, arterial blood gas analysis was performed on all participants at admission to assess pH, base excess, serum lactate, and bicarbonate levels as part of the baseline metabolic profile.

Randomization was performed using a computer-generated randomization sequence with allocation concealment by opaque sealed envelopes. Eligible neonates were randomized into two groups, a norepinephrine group and a dopamine group. Norepinephrine infusion was prepared by diluting 0.3 mg/kg of norepinephrine in 50 mL normal saline and initiated at 0.2 mcg/kg/min intravenously under continuous cardiorespiratory monitoring [11]. Dopamine infusion was prepared by diluting 30 mg/kg dopamine in 50 mL normal saline and initiated at 10 mcg/kg/min intravenously under continuous monitoring [8].

Reversal of shock was defined as mean blood pressure above the 5th percentile for gestational age, CRT over three seconds, reduction in heart rate by >10 beats/minute from baseline.

Monitoring and follow-up

After initiation of vasoactive infusion, neonates were assessed at 15, 30, and 45 minutes for heart rate, blood pressure, and CRT.

In the norepinephrine group, infusion rate was increased to 0.3 mcg/kg/min if shock persisted after 15 minutes and further increased to 0.4 mcg/kg/min if shock persisted after 30 minutes. In the dopamine group, infusion rate was increased to 15 mcg/kg/min after 15 minutes if shock persisted and further increased to 20 mcg/kg/min after 30 minutes if shock persisted. If shock persisted after 45 minutes despite escalation of therapy, adrenaline infusion was added and two-dimensional echocardiography was performed to guide further management.

Outcome variables

The primary outcome in our study was reversal of shock within 45 minutes of vasoactive therapy initiation, whereas secondary outcomes like requirement of additional vasoactive agents, need for mechanical ventilation, all-cause mortality by 28 days of life, occurrence of IVH, NEC, BPD, and ROP before discharge were recorded.

Sample size calculation

Sample size was calculated based on a previous study by Baske et al. [14], which observed a shock reversal rate of 30% in the dopamine group. We hypothesized that norepinephrine would yield a shock reversal rate of 60%. To detect this expected difference with a two-sided type I error (α) of 5%, a power of 90%, and an allocation ratio of 1:1, we calculated a required sample size of 40 neonates per group. After accounting for a 10% dropout rate, the final target sample size was set at 90 neonates, with 45 participants in each study arm.

Statistical analysis

Continuous variables were expressed as mean ± standard deviation for normally distributed data and median (interquartile range) for skewed data. Categorical variables were expressed as frequency and percentage.

Between groups, continuous variables were compared using the independent samples t-test. Categorical variables were analyzed using the chi-square test or Fisher’s exact test as appropriate. A p-value <0.05 was considered statistically significant. Statistical analysis was performed using SPSS version 25 (IBM Corp., Armonk, NY, USA).

## Results

**Figure 1:**
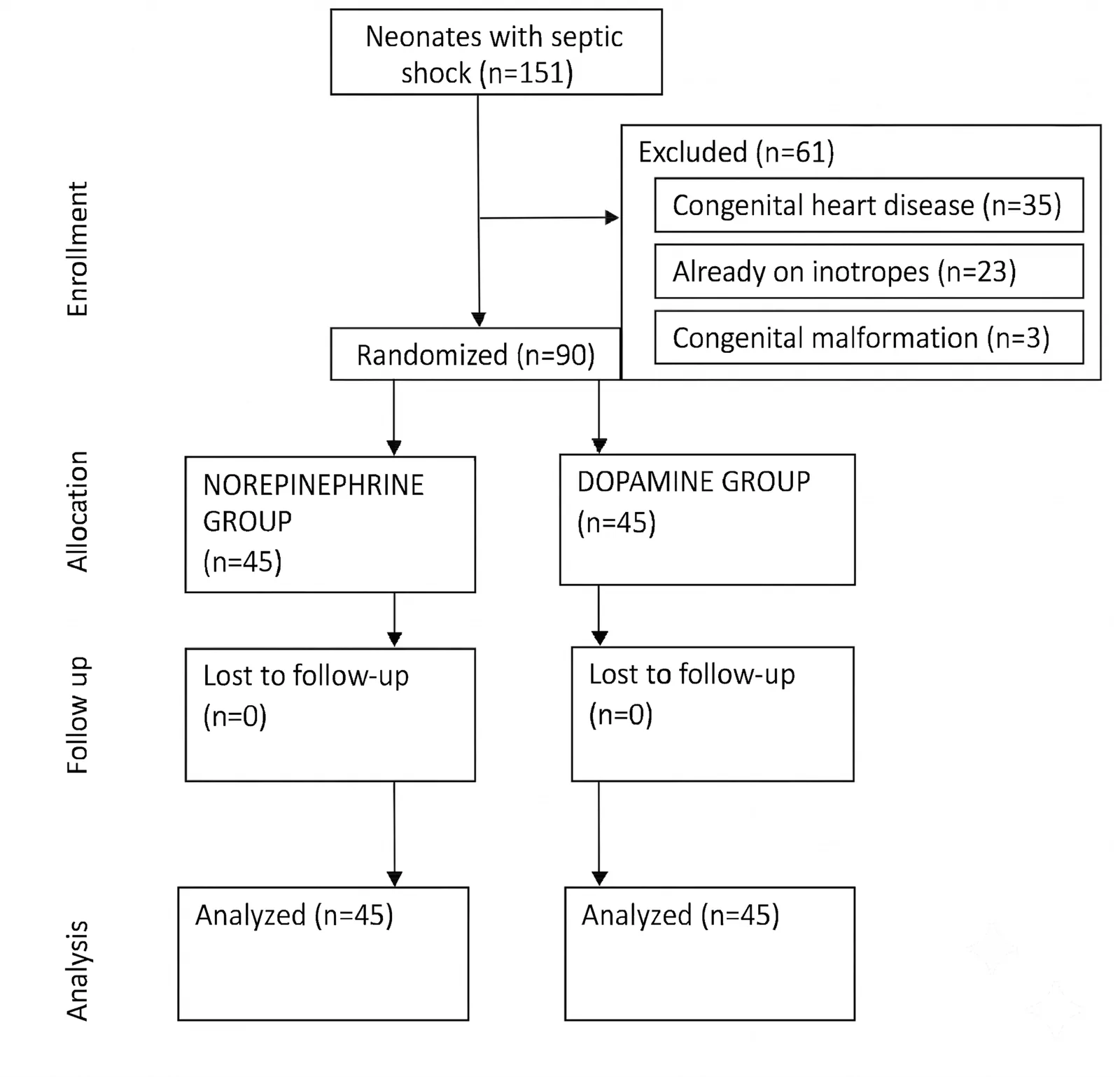
The Consolidated Standards of Reporting Trials (CONSORT) Flow Diagram

During the study period 151 neonates were admitted with shock, out of which 61 were excluded and 45 neonates were included in each group. Norepinephrine was given in one group and dopamine in the other group (Figure 1).

Baseline demographic characteristics were comparable between the two cohorts (Table 1). The majority of neonates in both groups were male (53.3% in the norepinephrine group vs. 55.6% in the dopamine group) and had a birth weight of ≤1.5 kg (33.3% vs. 35.6%, respectively). The mean gestational age was 34.70 ± 3.56 weeks in the norepinephrine arm and 34.65 ± 2.99 weeks in the dopamine arm. It was observed that the majority of neonates in both groups - 39 (86.7%) in the norepinephrine group and 34 (75.6%) in the dopamine group - had Apgar scores of 7-10. None of the neonates in the study had severe depression according to Apgar score. Antenatal steroids were administered among 14 (31.1%) mothers of neonates in the norepinephrine group and among 18 (40%) mothers of neonates in the dopamine group. PROM was present in 12 (26.6%) antenatal mothers in the norepinephrine and 10 (22.2%) in the dopamine group (Table 1).

**Table 1:** Baseline Characteristics of Neonates in the Norepinephrine and Dopamine Groups Data are presented as n (%). *P value<0.05 is significant. Abbreviations: Apgar: Appearance, Pulse, Grimace, Activity, and Respiration; LSCS: Lower Segment Caesarean Section; NVD: Normal Vaginal Delivery; SD: Standard Deviation.

Variable	Category	Norepinephrinene group (n=45) n (%)	Dopamine group (n=45) n (%)	Statistical test used	Test statistic	P value
Sex	Male	24 (53.3)	25 (55.6)	Chi-square test	χ² = 0.045 (df=1)	0.83
	Female	21 (46.7)	20 (44.4)			
Birth weight (kg)	≤1.5	15 (33.3)	16 (35.6)	Chi-square test	χ² = 5.751 (df=4)	0.18
	1.5-2.0	11 (24.4)	4 (8.9)			
	2.0-2.5	9 (20.0)	15 (33.3)			
	2.5-3.0	6 (13.3)	8 (17.8)			
	>3.0	4 (8.9)	2 (4.4)			
Gestational age	Extreme preterm (<28 weeks)	1 (2.2)	1 (2.2)	Chi-square test	χ² = 6.103 (df=4)	0.12
	Early preterm (28-31+6 weeks)	7 (15.6)	7 (15.6)			
	Moderate preterm (32-33+6 weeks)	7 (15.6)	6 (13.3)			
	Late preterm (34-36+6 weeks)	9 (20.0)	19 (42.2)			
	Term (≥37 weeks)	21 (46.7)	12 (26.7)			
Mode of delivery	NVD	19 (42.2)	15 (33.3)	Chi-square test	χ² = 0.756 (df=1)	0.39
	LSCS	26 (57.8)	30 (66.7)			
Apgar score	Severe depression (0-3)	0 (0.0)	0 (0.0)	Fisher's exact test		0.18
	Mild depression (4-6)	6 (13.3)	11 (24.4)			
	No depression (7-10)	39 (86.7)	34 (75.6)			

The distribution of early-onset sepsis (EOS) and late-onset sepsis (LOS) showed no statistically significant difference, with LOS being more prevalent in both the norepinephrine (66.7%) and dopamine (57.8%) groups (p = 0.38). Furthermore, the rate of culture-proven sepsis remained consistent across cohorts. Blood culture positivity was identified in 22.2% of the norepinephrine group and 15.6% of the dopamine group (p = 0.42), while CSF culture positivity was observed in 17.8% and 13.3%, respectively (p = 0.56) (Table 2).

**Table 2:** Sepsis Characteristics among the Study Population Data are presented as n (%). *P value<0.05 is significant. Abbreviations: CSF, cerebrospinal fluid.

Variable	Category	Norepinephrine group (n=45) n (%)	Dopamine group (n=45) n (%)	Statistical test used	Test statistic	P value
Clinical sepsis	Early-onset sepsis	15 (33.3)	19 (42.2)	Chi-square test	χ² = 0.756 (df=1)	0.38
	Late-onset sepsis	30 (66.7)	26 (57.8)			
Culture Positive	Blood culture	10 (22.2)	7 (15.6)	Chi-square test	χ² = 0.653 (df=1)	0.42
	CSF culture	8 (17.8)	6 (13.3)	Chi-square test	χ² = 0.338 (df=1)	0.56

The reversal of shock was assessed at 45 minutes following the initiation of vasoactive drug infusion. In the dopamine group, 16 neonates (35.6%) achieved shock reversal, compared to 14 neonates (31.1%) in the norepinephrine group. This slight difference was not statistically significant (p = 0.65), indicating comparable initial efficacy between the two interventions for restoring hemodynamic stability (Table 3).

**Table 3:** Comparison of Hemodynamic Parameters Between the Norepinephrine and Dopamine Groups Data are presented as mean ± standard deviation (SD) or n (%), as appropriate. Continuous variables were compared using the Independent Student's t-test, whereas categorical variables were compared using the Chi-square test when appropriate. Statistically significant p-values (P <0.05) Abbreviations: SBP: Systolic Blood Pressure; DBP: Diastolic Blood Pressure; CRT: Capillary Refill Time; SD: Standard Deviation.

Time of assessment	Parameter	Norepinephrine group (n=45)	Dopamine group (n=45)	Statistical test used	Test statistic	P value	
Baseline	SBP (mmHg)	44.84 ± 4.53	47.51 ± 5.06	Independent Student's t-test	t = -2.637 (df=88)	0.01	
	DBP (mmHg)	32.22 ± 4.04	33.40 ± 4.25	Independent Student's t-test	t = -1.350 (df=88)	0.18	
	Heart rate (beats/min)	134.20 ± 6.20	133.53 ± 5.50	Independent Student's t-test	t = 0.542 (df=88)	0.59	
15 min	SBP (mmHg)	47.98 ± 5.23	48.07 ± 5.07	Independent Student's t-test	t = -0.083 (df=88)	0.93	
	DBP (mmHg)	34.91 ± 5.25	33.09 ± 3.98	Independent Student's t-test	t = 1.853 (df=88)	0.07	
	Heart rate (beats/min)	136.00 ± 6.65	138.56 ± 17.68	Independent Student's t-test	t = -0.909 (df=88)	0.28	
	CRT <3 sec	25 (55.6)	27 (60.0)	Chi-square test	χ² = 0.182 (df=1)	0.67	
	CRT >3 sec	20 (44.4)	18 (40.0)				
30 min	SBP (mmHg)	49.93 ± 5.06	50.13 ± 4.91	Independent Student's t-test	t = −0.190 (df=88)	0.85	
	DBP (mmHg)	36.44 ± 5.64	34.29 ± 5.17	Independent Student's t-test	t = 1.885 (df=88)	0.07	
	Heart rate (beats/min)	140.76 ± 18.12	138.64 ± 5.79	Independent Student's t-test	t = 0.748 (df=88)	0.47	
	CRT <3 sec	25 (55.6)	27 (60.0)	Chi-square test	χ² = 0.182 (df=1)	0.67	
	CRT >3 sec	20 (44.4)	18 (40.0)				
45 min	SBP (mmHg)	52.07 ± 5.84	52.09 ± 5.59	Independent Student's t-test	t = −0.017 (df=88)	0.99	
	DBP (mmHg)	36.69 ± 5.60	34.91 ± 4.93	Independent Student's t-test	t = 1.600 (df=88)	0.11	
	Heart rate (beats/min)	138.73 ± 17.50	139.71 ± 6.13	Independent Student's t-test	t = −0.355 (df=88)	0.73	
	CRT <3 sec	26 (57.8)	27 (60.0)	Chi-square test	χ² = 0.046 (df=1)	0.83	
	CRT >3 sec	19 (42.2)	18 (40.0)				
	Reversal of shock present	14 (31.1)	16 (35.6)	Chi-square test	χ² = 0.200 (df=1)	0.65	
	Reversal of shock absent	31 (68.9)	29 (64.4)				

Table 4 shows that upon admission, acid-base status showed some variability between groups. While initial lactate levels (5.00 ± 1.30 mmol/L vs. 5.38 ± 1.34 mmol/L, p=0.177) and bicarbonate concentrations (21.76 ± 2.94 mEq/L vs. 21.18 ± 3.16 mEq/L, p=0.372) were comparable, a statistically significant difference was observed in base excess (6.98 ± 2.43 vs. 4.62 ± 7.23, p=0.043) and arterial pH (7.34 ± 0.06 vs. 7.33 ± 0.05, p=0.184).

**Table 4:** Comparison of Acid–Base Characteristics at Admission *P value<0.05 is significant. Data are presented as mean ± SD. Abbreviations: HCO₃⁻: bicarbonate.

Variable	Norepinephrine group (n=45)	Dopamine group (n=45)	Statistical test used	Test statistic	P value
Base excess	6.98 ± 2.43	4.62 ± 7.23	Independent Student's t-test	t = 2.076 (df=88)	0.043
Lactate (mmol/L)	5.00 ± 1.30	5.38 ± 1.34	Independent Student's t-test	t = -1.365 (df=88)	0.177
pH	7.34 ± 0.06	7.33 ± 0.05	Independent Student's t-test	t = 0.859 (df=88)	0.184
HCO₃⁻ (mEq/L)	21.76 ± 2.94	21.18 ± 3.16	Independent Student's t-test	t = 0.901 (df=88)	0.372

Similarly, all-cause mortality, requirement of additional vasoactive drugs and mechanical ventilation were higher in the norepinephrine group, but it was statistically insignificant. In our preterm study populations medium-term complications like IVH, NEC, ROP and BPD were found to be slightly increased in frequency in the norepinephrine group compared to the dopamine group but were not statistically significant. In our study, tachyarrhythmia was observed in four neonates (8.9%) in the norepinephrine group and five neonates (11.1%) in the dopamine group (p = 1.00). It is important to note that distinguishing drug-induced arrhythmias from those caused by the underlying pathophysiology of neonatal septic shock remains clinically challenging, as such complications are frequently inherent to the sepsis cascade itself rather than strictly adverse effects of the infused medications. Among the preterm populations in this study, medium-term morbidities - including IVH, NEC, ROP, and BPD - showed a slight, non-significant increase in frequency within the norepinephrine group (Table 5).

**Table 5:** Comparison of Clinical Outcomes Between the Study Groups *P value<0.05 is significant. Data are presented as n (%).

Variable	Norepinephrine group (n = 45) n (%)	Dopamine group (n = 45) n (%)	Statistical test	Test statistic	P value
Additional vasoactive drugs	11 (24.4)	10 (22.2)	Chi-square test	χ² = 0.062 (df = 1)	0.80
Tachyarrhythmia	4 (8.9)	5 (11.1)	Fisher's exact test	-	1.00
Mechanical ventilation	16 (35.6)	14 (31.1)	Chi-square test	χ² = 0.200 (df = 1)	0.65
All-cause mortality	7 (15.6)	6 (13.3)	Chi-square test	χ² = 0.090 (df = 1)	0.76
Intraventricular hemorrhage (IVH)	6 (13.3)	5 (11.1)	Fisher's exact test	-	1.00
Necrotizing enterocolitis (NEC)	3 (6.7)	2 (4.4)	Fisher's exact test	-	1.00
Retinopathy of prematurity (ROP)	8 (17.8)	7 (15.6)	Chi-square test	χ² = 0.078 (df = 1)	0.78
Bronchopulmonary dysplasia (BPD)	4 (8.9)	4 (8.9)	Fisher's exact test	-	1.00

## Discussion

Neonatal septic shock continues to be an important cause of mortality and morbidity, particularly among preterm and low birth weight neonates [1,3]. The primary objective of this study was to compare the efficacy of norepinephrine and dopamine as initial vasoactive agents in neonates with septic shock. Our results demonstrated comparable efficacy between the two groups, with no statistically significant difference in the rate of shock reversal at 45 minutes (31.1% vs. 35.6%, p=0.65). These findings indicate that both agents successfully restore hemodynamic stability in the acute phase of neonatal septic shock. While norepinephrine is increasingly favored in adult and pediatric critical care for its potential to improve systemic vascular resistance without the same risk of tachycardia associated with dopamine, our trial suggests that in a neonatal tertiary care setting, both agents remain viable options for initial hemodynamic resuscitation, provided they are accompanied by comprehensive supportive care.

In both neonatal groups the demographic characteristics were largely comparable. A predominance of male neonates and very low birth weight infants was observed, which is similar to findings reported in previous neonatal sepsis studies [14,20]. Late-onset sepsis was more common than early-onset sepsis in our cohort, suggesting a predominance of hospital-acquired infections, which has also been described in previous NICU-based studies [21].

Hemodynamic parameters, i.e., systolic blood pressure, diastolic blood pressure, and CRT, improved progressively in both groups without statistically significant intergroup differences. Similar findings were reported in recent neonatal studies evaluating vasoactive agents in septic shock [11,14,15].

Norepinephrine exerts its primary effects through α1-mediated vasoconstriction leading to increased systemic vascular resistance, while dopamine has dose-dependent inotropic and vasoconstrictive effects [9,11]. Although norepinephrine theoretically offers superior vascular support in distributive shock, this pharmacological advantage did not translate into significantly improved early shock reversal in our study. Similar observations have been reported in pediatric septic shock studies [5,15].

The requirement for additional vasoactive support, need for mechanical ventilation, and all-cause mortality were comparable between both groups. These findings are consistent with previous literature demonstrating that overall outcomes in neonatal septic shock are multifactorial and depend on timely diagnosis, antibiotic administration, adequate fluid resuscitation, and supportive intensive care measures in addition to vasoactive therapy [5,22].

The incidence of short-term complications such as IVH, NEC, BPD, and ROP was also comparable between groups. Previous studies evaluating vasoactive agents in neonates have similarly reported no significant differences in neonatal morbidities among different vasoactive therapies [14].

Although the pH difference did not reach statistical significance, the variance in base excess suggests that neonates in the norepinephrine group may have presented with a marginally different degree of metabolic buffering capacity at baseline. However, given that the primary markers of tissue hypoperfusion - specifically lactate and bicarbonate - were statistically similar, these minor imbalances in acid-base status are unlikely to represent a fundamental divergence in the severity of shock between the two cohorts at the time of enrollment. Previous studies have shown that lactate clearance correlates with improved tissue perfusion and clinical outcomes in septic shock [22].

Tachyarrhythmias were observed in both groups, although the incidence was slightly higher in the dopamine group. Dopamine has previously been associated with increased risk of tachyarrhythmias due to β-adrenergic stimulation [9]. This multifactorial nature of arrhythmia in the critically ill neonate makes it challenging to definitively attribute causality to the choice of vasoactive agent.

The primary strength of this study lies in its randomized controlled design, which minimizes selection bias and ensures that the baseline characteristics of the norepinephrine and dopamine cohorts were well-balanced. By focusing on clinically relevant hemodynamic outcomes, such as shock reversal time, vital sign stabilization, and end-organ perfusion markers, the study provides pragmatic, actionable data for neonatal intensive care providers.

However, certain limitations of our study should be acknowledged like its single-center design, relatively small sample size, lack of blinding, absence of advanced hemodynamic monitoring (such as functional echocardiography), lack of data on length of hospital stay and long-term neurodevelopmental follow-up.

Further large multicentric studies incorporating bedside functional echocardiography and individualized hemodynamic assessment are required to determine the optimal vasoactive strategy in neonatal septic shock.

## Conclusions

Norepinephrine and dopamine demonstrated comparable efficacy in achieving early reversal of neonatal septic shock, with insignificant differences in mortality or short-term complications. These findings suggest that both agents can be used as initial vasoactive therapy in neonatal septic shock. However, given the evolving evidence favoring norepinephrine for its hemodynamic profile and vasoconstrictive properties, larger multicentric randomized trials are warranted to establish definitive guidelines and individualized physiology-based vasopressor strategies in this vulnerable population.

## References

[1] Shane AL, Sánchez PJ, Stoll BJ (2017). Neonatal sepsis. Lancet.

[2] Wynn JL, Wong HR (2010). Pathophysiology and treatment of septic shock in neonates. Clin Perinatol.

[3] Fleischmann-Struzek C, Goldfarb DM, Schlattmann P (2018). The global burden of pediatric and neonatal sepsis. Lancet Respir Med.

[4] Singer M, Deutschman CS, Seymour CW (2016). The third international consensus definitions for sepsis and septic shock (Sepsis-3). JAMA.

[5] Goldstein B, Giroir B, Randolph A (2005). International pediatric sepsis consensus conference: definitions for sepsis and organ dysfunction in pediatrics. Pediatr Crit Care Med.

[6] Schlapbach LJ, Watson RS, Sorce LR (2024). International consensus criteria for pediatric sepsis and septic shock. JAMA.

[7] Davis AL, Carcillo JA, Aneja RK (2017). American College of Critical Care Medicine clinical practice parameters for hemodynamic support of pediatric and neonatal septic shock. Crit Care Med.

[8] Mazhari MY, Priyadarshi M, Singh P, Chaurasia S, Basu S (2025). Norepinephrine versus dopamine for septic shock in neonates: a randomized controlled trial. J Pediatr.

[9] Subhedar NV, Shaw NJ (2000). Dopamine versus dobutamine for hypotensive preterm infants. Cochrane Database Syst Rev.

[10] Ventura AM, Shieh HH, Bousso A (2015). Double-blind prospective randomized controlled trial of dopamine versus epinephrine as first-line vasoactive drugs in pediatric septic shock. Crit Care Med.

[11] Tourneux P, Rakza T, Abazine A, Krim G, Storme L (2008). Noradrenaline for management of septic shock refractory to fluid loading and dopamine or dobutamine in full-term newborn infants. Acta Paediatr.

[12] Gupta S, Agrawal G, Thakur S, Gupta A, Wazir S (2022). The effect of norepinephrine on clinical and hemodynamic parameters in neonates with shock: a retrospective cohort study. Eur J Pediatr.

[13] Kallimath A, Garegrat R, Patnaik S, Singh Y, Soni NB, Suryawanshi P (2024). Hemodynamic effects of noradrenaline in neonatal septic shock: a prospective cohort study. J Trop Pediatr.

[14] Baske K, Saini SS, Dutta S, Sundaram V (2018). Epinephrine versus dopamine in neonatal septic shock: a double-blind randomized controlled trial. Eur J Pediatr.

[15] Singh R, Bandyopadhyay T, Maria A (2025). Dopamine versus norepinephrine in fluid-refractory septic shock among preterm neonates: a double-blind randomized controlled trial. J Trop Pediatr.

[16] Wen L, Xu L (2020). The efficacy of dopamine versus epinephrine for pediatric or neonatal septic shock: a meta-analysis of randomized controlled studies. Ital J Pediatr.

[17] Gupta T (2022). Cerebrospinal fluid analysis in infectious disease. Pediatr Infect Dis.

[18] Ballard JL, Novak KK, Driver M (1979). A simplified score for assessment of fetal maturation of newly born infants. J Pediatr.

[19] Apgar V (1953). A proposal for a new method of evaluation of the newborn infant. Curr Res Anesth Analg.

[20] Stoll BJ, Hansen NI, Sánchez PJ (2011). Early onset neonatal sepsis: the burden of group B Streptococcal and E. coli disease continues. Pediatrics.

[21] Kaffe K, Syrogiannopoulos GA, Petinaki E, Goudesidou M, Kalaitzi A, Gounaris A, Grivea IN (2025). Epidemiology and outcomes of late-onset neonatal sepsis in preterm infants in a tertiary hospital. Children (Basel).

[22] Levy MM, Evans LE, Rhodes A (2018). The Surviving Sepsis Campaign Bundle: 2018 update. Intensive Care Med.

